# Students’ engagement and perceptions of small group tutorial classes among undergraduate medical students

**DOI:** 10.30476/jamp.2020.86925.1280

**Published:** 2021-01

**Authors:** SHIVANANDA NAYAK B, VINEETH SRIDEVI, PRADEEPKUMAR SAHU, NAGENDRA SUBBARAMAIAH, LATHA TELANG, PRASHANTHKUMAR GOUDAPPALA, CHANDRAKANTH HALAPPA KARIAPPA, AKANKSHA SHARMA, MAANASA SAGARI K, AKASH S NAYAK

**Affiliations:** 1 Subbaiah Institute of Medical Sciences, Purle, Shivamogga, Karnataka, India; 2 Faculty of Medical Sciences, The University of the West Indies, St Augustine, Trinidad and Tobago; 3 Kasturba Medical College, Manipal Academy of higher education, Manipal, Karnataka, India

**Keywords:** Faculty, Perception, Tutorial, Teaching

## Abstract

**Introduction:**

Small group teaching is an educational strategy that may be used to facilitate learning. Tutorials enable an adult approach toward learning where students take responsibility for their own learning. We aimed to investigate the students’ engagement and perceptions of small group tutorial classes among undergraduate medical students.

**Methods:**

A cross‑sectional, descriptive survey was conducted at the Subbaiah Institute of Medical Sciences, where we collected the data from 300 undergraduate students using convenience sampling method. A self‑administered questionnaire consisting of 22 items which was piloted on 20 students, and six experienced medical educators were consulted for face validation. The internal consistency of the questionnaire measured by Cronbach’s alpha reliability test was 0.80. It was used to measure the students’ perception on the effectiveness of tutorials with regard to learning experience, teamwork, confidence, communication skills, and role of the teacher. Statistical analyses included mean and standard deviation for the description of each item, t-test to compare the mean scores for gender and class year, and one‑way analysis of variance between groups for age group comparisons using SPSS version 24 software.

**Results:**

Students’ overall perceptions of small group teaching effectiveness showed that tutorials were beneficial to their learning process (mean: 3.61±0.50). The majority of the students have positive perceptions toward small group effectiveness, particularly in learning experience (mean: 3.72±0.68) and teamwork (mean: 3.36±0.59). A significant difference was found between year 1 and year 2 students with regards to learning experience (p<0.001), teamwork (p<0.05), communication skills (p<0.05), and the role of the tutor (p<0.001). Additionally, the mean scores, measuring overall effectiveness of tutorials, for the 2nd year students were significantly higher than that for the 1^st^ year students (3.70± .41 and 3.50 ±0.57, (p<0.001).

**Conclusions:**

The data of this study show that tutorial is an effective small group teaching method for medical students compared to large group teaching.

## Introduction

Small group tutorial teaching has become an increasingly important and necessary component of undergraduate medical education. Relatively little is known about the students’ perceptions of small group goals, effective teaching practices, and methods of evaluation in the small group settings despite the increased use of small group tutorial teaching in medical education ( 1 ).

Small group tutorial teaching has increasingly been adopted by many medical schools and other disciplines as well, keeping pace with the technological advancement in the last five decades and changing career demands ( 2 ).

In India, there are more than 535 recognized medical colleges and a large number of medical students graduating annually. The medical college lecturers face the challenge of teaching large classes while maintaining and improving the quality of medical education. Currently, didactic lectures are the method of choice to get across a large amount of theoretical information to a large group of learners at one time. Hence, most of the students see knowledge as something to be transmitted by the teacher to them. Teaching in many countries is dominated by teacher-centred classrooms ( 3 ). Some of the important concepts like flexibility in learning, problem solving, critical thinking, and independent learning are least recognized ( 4 ). The best solution for eradicating teacher-centred teaching is to involve the students actively in the learning process, and small group teaching, like tutorials and case-based learning, is optimal for this. 

Small group teaching is an educational strategy that may be used to facilitate learning. Small group learning has grown in popularity in medical education as it offers a dynamic and collaborative setting for learning ( 5 ). Tutorials enable an adult approach toward learning where students take responsibility for their own learning ( 6 ). A recent review of motivational and cognitive effects of tutorials ( 7 ) showed that students fostered interactive learning and positive cognitive effects, such as recall of information and cognitive conflicts leading to conceptual change ( 8 , 9 ). Tutorials have a direct positive effect on children and motivate them to learn.

This type of small group teaching plays a pivotal role in the overall growth of students, whether it is a tutorial, seminar, simulated learning, problem‑based learning, or case- based learning. Tutorial is a popular effective learning approach conceived and implemented in education to enhance the students’ application of knowledge and higher‑order thinking skills ( 10 ). In tutorials, students get the opportunity to discuss the issues, ask questions, reflect critically, clarify misunderstandings, test hypotheses, and evaluate ideas while closely interacting with the teacher and other students.

Working in small groups make the students active, confident, and independent in their learning ( 11 ). The tutorials make it possible for students to support each other in the problem‑solving process and provide a means to “scaffold” the learning process of the student ( 3 ). Students feel comfortable to express their thoughts and ideas clearly. They can reflect on their experiences while learning from their peers ( 12 ). Small group teaching increases the students’ interest in learning, provides the opportunity to clarify the points of confusion, promotes student‑faculty and peer‑peer interaction, enhances teamwork ability, and fosters communication skills ( 13 , 14 ). In addition, small group teaching is useful in promoting higher‑level intellectual skills such as reasoning, problem‑solving, and critical thinking. These skills are also important for medical students who will eventually become involved professionally with patients and other health‑care professionals ( 15 ).

A number of publications have reported the students’ perceptions of effective tutors in problem-based learning curricula ( 10 , 16 ). However, relatively little is known about perceptions of students on the effectiveness of tutorial as a small group teaching. The purpose of the study was to measure the perceptions of medical students on the effectiveness of tutorial among undergraduate medical students.

## Methods

For the present cross-sectional study, we conducted a descriptive survey at the Subbaiah Institute of Medical Sciences (SIMS), Shivamogga affiliated to Rajiv Gandhi University, India. We collected the data from year 1 and year 2 medical undergraduate students. We collected the data from 300 undergraduate students using convenience sampling method ( 17 ). 

Faculty members from SIMS with an experience of tutorials and knowledge of research developed the questionnaire. A pilot test of the questionnaire was done on 20 undergraduate medical students each from first year and second year. Their responses and feedback were used to assess the format, language, and clarity of the items. Based on the feedback given by the students and after consultation with 6 faculty members, necessary revisions of the questionnaire were made. The 22 items of the questionnaire were scored on a 5‑point Likert scale ranging from “strongly agree,” [4] to “strongly disagree” [0] which measure the students’ perception of small group tutorial effectiveness. The average score for each participant was calculated on the 5-point scale. The questionnaire comprises 5 domains. The average of the first 5 items measured the students’ perception towards learning experience. Each of the second, third and fourth parts consisted of 4 items and the average of those items measured the students’ perception towards teamwork, confidence, and communication skills. The average of the last five items measured the students’ perception towards the role of the tutor.

The internal consistency of all 22 items measured by Cronbach’s alpha reliability test was 0.80. We included open‑ended questions in the questionnaire to get response from the students on the challenges and benefits of small group tutorials.

The questionnaire was filled by the students during 2019-2020 academic year. The purpose of the study was to inform to the participants. Participation was voluntary, and all participants remained anonymous. Ethical approval for the study was obtained from the ethics committee of Subbaiah Medical Sciences Institute.

We used SPSS version 24 software (IBM Corporation, New York, USA) to analyze the collected data. The continuous variables were summarized as mean and standard deviation for each item of the questionnaire. Student t‑tests were used to analyse the differences between the perception of students on the effectiveness of tutorials with respect to gender and year of the study (year 1 and 2). To compare the impact of age on the effectiveness of tutorial, we used a one‑way analysis of variance (ANOVA). P <0.05 was considered statistically significant for both t‑tests and ANOVA. Additionally, we did qualitative analysis based on the responses from the open-ended questions given by students regarding the benefits and challenges of tutorials.

## Results

Demographic characteristics of the students are shown in Table 1. A total of 136 first year and 164 second year medical students completed the questionnaires. Of these respondents, 133 (44.33%) were male and 167 (55.67%) were female. The mean age of the respondents was 20 years, with a range of 19-24 years. A large percentage of respondents was 19 years old or younger (70.67 %) and 20 years (20.33%). 

**Table 1:** Demographic information of the participants

Participants characteristics	Frequency	Percentage
Gender		
Male	133	44.33
Female	167	55.67
Current class		
Year 1	136	45.33
Year 2	164	54.65
Age		
19 or younger	212	70.67
Only 20	61	20.33
21 or older	27	9.00

The overall response of the students showed that the tutorial sessions were found to be beneficial to their learning different concepts (mean: 3.61±0.50). Table 2 shows that out of the 5 components of the small group tutorials, students scored highest (mean: 3.72±0.68) on “learning experiences” and “communication skill” (3.72±0.69), whereas they scored lowest (mean: 3.36±0.59) on “team work”. The mean score for the 22 items varied between 2.68 and 4.22. The highest scoring items were for “It becomes easier to learn when members of the group share their thoughts, ideas and information” (4.22±0.85); “Tutor encouragement” (4.06±0.92) and “learning in tutorial helped me improve my ability to think and solve problems rather than just memorize information” (3.96±0.084). Items with lowest mean scores were “My group members made me feel as though I was not as smart as they were” (2.42±1.33) and “I felt nervous when I was asked to express my thoughts in a group.” (2.68±1.34).

**Table 2:** Item wise average scores for the students’ perception on tutorial sessions

Small Group Effectiveness Factors	Mean±SD
Learning experiences	3.72±0.68
1. Discussions held in tutorial helped in understanding the subject better.	3.76±0.85
2. Learning in tutorial helped me improving my ability to think and solve problems rather than just memorizing information.	3.96±0.84
3. The activities of tutorial taught me life-long learning.	3.31±1.21
4. Tutorial sessions led me to deep and active learning.	3.66±0.99
5. The knowledge and skills acquired in tutorial will help me in clinical practice.	3.95±1.05
Team work	3.36±0.59
1. The activities in tutorial helped me to develop skills on working as a member of a team.	3.49±1.16
2. My group members made me feel as though, I am not as smart as they are.	2.42±1.33
3. Group members were respectful to all the members.	3.32±1.05
4. It becomes easier to learn when members of the group share their thoughts, ideas and information.	4.22±0.85
Confidence	3.60±0.68
1. Small group tutorial made the learning more challenging, interesting, motivating, engaging and fun.	3.94±0.98
2. My interest in learning the subject increased while working in small tutorial group.	3.86±0.98
3. I felt nervous when I was asked to express my thoughts in a group.	2.68±1.34
4. Learning in small group tutorial motivated me to work hard and participate actively in the group activities.	3.93±0.98
Communication skills	3.72±0.69
1. Tutorial activities improved my ability to communicate effectively.	3.92±0.94
2. I listen more attentively to what other members talk in the group.	3.74±0.96
3. I feel easier to express doubts and feelings in a tutorial group.	3.66±1.09
4. I developed the ability to summarize the views of others.	3.57±1.05
Role of the tutor	3.61±0.71
1. Tutor in the group provided proper guidance for self-learning.	3.83±1.02
2. Tutor paid sufficient personal attention to the students during the tutorial session.	3.77±1.10
3. Tutor was talking a lot in some of the sessions.	2.85±1.24
4. Tutor encouraged all students including less involved students to take part in the discussion.	4.06±0.92
5. Tutor provided useful feedback on my progress.	3.54±1.12
Overall	3.61±0.50

We found no significant difference between the mean scores of male and female students on the learning experience, teamwork, confidence, communication skills, role of the tutor, and overall students’ perceptions. However, the mean scores of male students in each of the components were higher than their female counterparts (Table 3).

**Table 3:** Comparative scores for the students’ perceptions of tutorial effectiveness based on gender

Variables	Male	Female	t value	P (two tailed)
Learning Experiences	3.79±0.60	3.68±0.73	1.39	0.165
Team Work	3.37±0.61	3.36±0.57	0.16	0.870
Confidence	3.62±0.67	3.58±0.70	0.41	0.680
Communication Skills	3.78±0.70	3.68±0.69	1.33	0.185
Role of the Tutor	3.62±0.68	3.60±0.74	0.26	0.795
Overall	3.65±0.45	3.58±0.53	1.05	0.294

A significant difference was found between year 1 and year 2 students with regards to learning experience (p<0.001), teamwork (p<0.001), communication skills (p<0.05) and the role of the tutor (p<0.001). Additionally, the mean scores showed the overall effectiveness of tutorials (Table 4). 

**Table 4:** Comparative scores for the students’ perceptions of tutorial effectiveness according to the year of study

Variables	Year 1	Year 2	t value	P (two tailed)
Learning Experiences	3.57±0.76	3.85±0.57	3.57	<0.001
Team Work	3.27±0.62	3.44±0.54	2.58	0.010
Confidence	3.56±0.78	3.63±0.59	0.83	0.407
Communication Skills	3.60±0.75	3.82±0.63	2.81	0.005
Role of the Tutor	3.45±0.85	3.74±0.55	3.61	<0.001
Overall	3.50±0.57	3.70±0.41	3.67	<0.001

Furthermore, one‑way ANOVA between groups was conducted to compare the differences in each of the five components concerning the students’ perceptions of the effectiveness of tutorials and the students’ age (Table 5). The association between the students’ perception on the effectiveness of each factor of small group tutorial and age was not significant. In addition, p=0.502 displayed on the Table indicates that there were no significant differences (F=0.69) in the overall effectiveness of the tutorial score with regard to the age of the students.

**Table 5:** The one-way ANOVA between the groups according to age (N=195)

Variable	Subscales	LE	TW	Co	CS	RT	Overall
Age							
19 and younger	Mean±SD	3.68±0.71	3.35±0.60	3.60±0.71	3.74±0.67	3.56±0.73	3.59±0.50
Only 20	Mean±SD	3.81±0.57	3.35±0.59	3.60±0.60	3.66±0.74	3.70±0.68	3.63±0.50
21 and above	Mean±SD	3.90±0.59	3.46±0.45	3.63±0.71	3.69±0.77	3.81±0.63	3.71±0.47
P value		0.173	0.667	0.981	0.687	0.134	0.502

LE: Learning Experiences, TW: Team Work. Co: Confidence CS: Communication Skills, RT: Role of the Tutor

### What students say about tutorial class?

Along with the 22 close ended items, we also asked the students to respond if they were satisfied with the tutorial classes. Furthermore, we asked them to state the challenges they experienced from tutorial classes. Students found tutorial classes beneficial as follows:

### Advantages of tutorial classes

While the students were answering the questions, if they experienced any potential benefits from the tutorial classes, it was noted that they had expressed positive opinion towards the tutorial. They believed that small group tutorials were more engaging and interactive than the traditional large group classes. They believed that tutorial classes helped them clarify the concept in a simple manner. Moreover, it helped them improve their problem solving and life-long learning skills. It was stated by one of the students that:

“Small group tutorials helped me improve my ability to think and solve problems; It helped me a lot to understand the concepts clearly. It also helped me to remember and recollect the facts learnt in lectures and revisions. Tutorials provide a place for rectification of mistakes. Questions discussed in tutorials are useful for the exams.”

The majority of students considered tutorial as an effective teaching method that helped them increase their confidence level. Because the number of students was small in the tutorial, quieter and less participatory students got courage and motivation to take part in the learning process. A student who hesitated to speak in a large class said:

“It was a good experience for me. Now, I am not afraid to ask the group members or the tutor questions. I think I am confident now to express my ideas and share my thoughts with the group.”

In the tutorial classes, students discussed and solved the concept as a team. Students found out the problems and solutions together as a team taking guidance from the tutor. Each student respected the ideas and viewpoints of other students. One student stated: 

“I believe that learning together in a tutorial class is more effective and good for long term memory. I listened to others carefully and other students also respected my ideas. It was a good example of teamwork.”

Tutors in tutorial classes played a vital role by providing necessary support and guidance to the group members when they required. Because of the small number of students in a group, tutors gave proper attention to each student. Students were satisfied with the contribution of the tutors, making the group active and lively. They asked the students thoughtful questions to discuss the topics deeply with clear understanding. One of the students stated:

“I think the tutor’s role is important in the tutorial class. He not only encouraged equal participation in the group, but also provided constructive feedback to make the learning process useful and productive. Whenever we faced any difficulties on any issue, we took guidance from the tutor to address the problem. He also encouraged less involved students to take part in the discussion”. 

In tutorial classes, every student got chance to express his/her views. Students expressed their thought, listened to each other attentively and interacted to discuss the topics. This is a good platform to improve communication skills. On communication skill, one of the students said:

“Tutorial classes helped me to communicate effectively. Now I can express my thoughts without any fear. It has improved my interpersonal skills.” 

Smaller crowds allow for more efficient discussions. It is easier to consider the views of all active members. Tutorial classes helped the students to discuss the topic with friends and teachers. Tutorials provide a place for rectification of mistakes. 

### Challenges of Tutorial Classes

Despite the fact that tutorial classes are beneficial for students, we should not forget that no teaching strategy is free from criticism. Some students believed taking part in tutorial was a waste of time. Few students were well prepared because they were not aware about the topics to be discussed in the tutorial classes. One of the students stated:

“There was lack of time to discuss everything. It’s practically not possible to listen to everyone’s views and opinions. Discipline wasn’t maintained; it made the class noisy.”

 Some students were not comfortable at all because only few students in the group were speaking too much and dominating in the discussion process. Students who were less involved in the tutorial felt themselves neglected in the interactive process. Students whose involvement was minimal in the class did not get motivation to share and express their thoughts and viewpoints in front of the tutors and peer group. Many students were not confident enough to speak although they wanted to take part in the learning process. One student said: 

“A few members in the group talked too much, whereas other students did not talk; I felt nervous when I was asked to express my thoughts in a group.”

In a few occasions, one student was speaking for a long time which made the learning less interesting. Some students were stressful because they were not well prepared and had pressure to actively take part and contribute in the tutorial classes.

Tutors are an integral part of the tutorial classes. However, some students found tutor related issues during the learning process. Tutors did not emphasize equal participation in the class and thus only a few students played dominating role in the discussion. Those students, whose involvement was minimal, were expecting motivation and encouragement from the tutors. They believed that tutors should be informed about the topic well before the time and give adequate time to prepare the topic.

## Discussion

 Various teaching methodologies have come into play for benefit of the students to improve their learning and thereby their performance in the board and competitive examinations. Pre-clinical subjects of the medical curriculum are taught mostly by didactic lectures, practical classes, “tutorials” and problem--based learning in India. Most of the teaching is teacher centred where minimum active participation of the student is seen. Nowadays, student-centred approaches have gained much recognition and there is a pragmatic shift towards this approach in accordance with “S” of SPICES model by Harden et al., ( 18 ) where “S” stands for a student-centred approach.

The evaluation of the effectiveness of these teaching methodologies can be achieved by a constant informal communication with the students in the form of designed questionnaires. Therefore, the present study was designed in the form of a questionnaire with the objective to elicit the varied perceptions of the students regarding the extent to which they benefitted from the tutorials as a teaching-learning method and the utility of the prevailing system of teaching.

The mean scores of most of the statements were close to four (on a 5‑point scale). This indicates that small group tutorials have a positive and significant influence on the students’ educational perception. Several studies reported that small group teaching sessions had positive effects on the students’ learning ( 19 , 20 ). In our further investigation, no significant difference was seen in the students’ perception on the effectiveness of small group tutorials with respect to the age of the student, gender and academic year; this reveals that irrespective of the gender, year of study and age, the students have similar perception on small group tutorials. 

A number of studies have demonstrated that small group tutorials help in enhancing problem‑solving skills, providing the opportunity to clarify the point of confusion, increasing understanding of the subject, improving self‑directing skills, developing critical thinking, and fostering active learning ( 21 ). In the current study, we have also noted similar findings on the students’ where they had a very positive learning experience. The students also commented that it helped them in understanding of the subject in depth which would help them in their future clinical practice.

One of the unique characteristics of small group tutorials is that it is a unique opportunity for the students to work as a team. Truly speaking, a collection of individuals is not a team until they work collectively, interact and listen attentively to one another, and respect the views of others. A small tutorial group motivates its members to exert maximum effort and help each other to create an effective learning environment. In the present study, we have found that small groups teaching provides similar avenue for the students to have collaborative learning and teamwork. Furthermore, the comments given by students reflect that they learned to respect one another’s point of view and work together for constructive learning.

It is important to recognize that tutorials bring in a great degree of confidence among the students. Similarly, in our study age, gender, and year of study had no significance in the increase in the students’ confidence level . However, the mean score of year 2 students on the students’ perception on confidence was slightly higher than that of year 1 students. This is primarily because the year 1 students are usually apprehensive and distant, but gradually they get more confident and move closer to these discussions and brain storming sessions. Thus, it clearly suggests that a time period is required for the students to get used to the way of working in small tutorial groups and how to cope with it. 

A number of studies have reported advantages for tutorials which include increasing student‑faculty and peer‑peer interaction, improving communication skills, increasing opportunities to ask questions, and improving presentation skills of the students ( 11 ). In our study, it was obvious that tutorials helped them to communicate effectively, but they were nervous when they were asked to express their ideas and thoughts. This may be primarily due to the talkative students who tried to dominate the group. Thus, the quieter students did less or no efforts to interact. In a study carried out by Rahman et al. ( 22 ), the authors have found in many cases that the students were not satisfied with working within a group and all members within a group did not participate equally in the discussions.

The tutors play a nondirective but a significant role in facilitating the small group sessions ( 23 ). In the present study, the mean scores for students’ perception about the role of the tutor range from 2.85 to 4.06. The lowest scoring item as we found is “Tutor was talking a lot in some of the sessions.” Further, the answer to the open‑ended questions confirms that students have expressed their dissatisfaction with the dominating role played by some of the tutors. An important implication of this finding is that faculty development should take place highlighting on how to and not to intervene the small group tutorial session. Instead of playing the dominating role, the tutor should motivate the leader to guide lead the group and encourage other members, including the quieter students, to take part actively in the learning process. Besides, the tutor should provide constructive feedback to the students at the end of each session because it contributes to their progress in learning throughout the medical program ( 24 ).

According to Tripati et al. ( 25 ), more students felt comfortable and were satisfied with the tutorial mode of teaching than with active learning strategies. This is confirmed by the fact that many studies have shown that small group teaching method is better for understanding different aspects of therapeutics like analyzing the clinical case scenario and applying clinical knowledge in writing prescriptions ( 26 ). Tutorials as a method of teaching learning is more interactive and specific than books or lectures.

Ananthkrishnan et al. ( 27 ) has also stressed the importance of microteaching session for teachers as a preparatory vehicle for imparting quality education. A similar finding has also been reported by Garg et al where 34.92% of the respondents opted for introduction of group discussion in the teaching programme ( 28 ). As indicated in a study by Advani et al., more students want clinically oriented lectures ( 29 ). 

## Conclusion

In recent years, the importance of small group teaching inclusive of tutorial sessions are being acknowledged. It has been considered to play as an integral part of the students’ long term learning. Tutorial can be more effective if the tutor focuses on active involvement of students in the learning process. The tutor’s motivation to quitter students will encourage them to engage in and contribute to constructing new knowledge in a more productive way. 
